# SWEF Proteins Distinctly Control Maintenance and Differentiation of Hematopoietic Stem Cells

**DOI:** 10.1371/journal.pone.0161060

**Published:** 2016-08-25

**Authors:** Tatsiana Ripich, Carlos Andrés Chacón-Martínez, Luise Fischer, Alessandra Pernis, Nadine Kiessling, Annette I. Garbe, Rolf Jessberger

**Affiliations:** 1 Institute of Physiological Chemistry, Technische Universität Dresden, 01307, Dresden, Germany; 2 Osteoimmunology, CRTD, Technische Universität Dresden, 01307, Dresden, Germany; 3 Autoimmunity and Inflammation Program, Hospital for Special Surgery, New York, NY, 10021, United States of America; Emory University, UNITED STATES

## Abstract

SWAP-70 and DEF6, two proteins that feature similar domain and motif arrangements, are mainly known for their functions in differentiated hematopoietic cells. Both proteins interact with and regulate RhoGTPases and F-actin dynamics, yet their role in hematopoietic stem and precursor cells (HSPCs) remained unexplored. Here, the role of the SWEF proteins SWAP-70 and DEF6 in HSPCs was examined. Both SWEF proteins are expressed in HSCs. HSCs and different precursor populations were analyzed in mice deficient for SWAP-70, DEF6, SWAP-70 and DEF6 (double knockout, DKO), and wild-type controls. HSPCs isolated from these strains were used for competitive adoptive transfer into irradiated wild-type mice. Reconstitution of the myeloid and lymphoid lineages in the recipient mice was determined. The numbers of HSPCs in the bone marrow of *Swap-70*^*-/-*^ and *Swap-70*^*-/-*^*Def6*^*-/-*^ mice were >3-fold increased. When transplanted into lethally irradiated wild-type recipients, the reconstitution potential of *Swap-70*^*-/-*^ HSPCs was intrinsically impaired in competing with wild-type HSPCs for contribution to hematopoiesis. *Def6*^*-/-*^ HSPCs show wild type-like reconstitution potential under the same transplantation conditions. DKO HSPCs reconstituted to only 25% of wild-type levels, indicating a partial rescue by DEF6 deficiency in the *Swap-70*^*-/-*^ background. Our study reveals the two SWEF proteins as important contributors to HSPC biology. Despite their similarity these two proteins regulate HSC/progenitor homeostasis, self-renewal, lineage contributions and repopulation in a distinct and mostly antagonistic manner.

## Introduction

SWAP-70 and DEF6 (also known as IBP or SLAT) are the only two members of the SWEF family. They have been described as a unique type of Rho-family GTPase and F-actin regulatory proteins [1–12]. From N- to C-terminus, both proteins feature a putative EF-hand Ca^2+^-binding domain, followed by a pleckstrin-homology (PH) domain, which is involved in membrane targeting through binding of phosphatidylinositol-3,4,5-triphosphate (PIP3), and a domain weakly homologous to Dbl homology (DH) domains. A hallmark of SWAP-70 and DEF6 is the arrangement of the PH-DH domains, which is the reverse of that observed in typical GEFs for Rho GTPases, wherein a C-terminal PH domain flanks the DH domain. SWAP-70 features an F-actin binding domain at its C-terminus, and both proteins carry several nuclear localization and a nuclear exit signal sequences. SWAP-70 was found to contribute to Rac activation *in vitro* and in hematopoietic cells and plays a role in specific processes during remodeling of the actin cytoskeleton thus being involved in cell adhesion and migration *in vitro* and *in vivo*. DEF6 has been characterized as a GEF for Cdc42 and Rac2 and is involved T cell receptor (TCR)-mediated cytoskeletal dynamics. Both proteins are expressed in several hematopoietic cell types, in many transformed cell lines and some tumor types. However, expression and role(s) in many progenitor cell types including in hematopoietic stem and precursor cells (HSPCs) have not been described.

Hematopoietic stem cells (HSCs), first described by Till and McCulloch [13] and purified and characterized by Weissman [14] serve to generate all lineages of the hematopoietic system. HSCs and progenitor cells have been very extensively studied. Still, however, regulators that determine the capacity of HSPCs to regenerate a deficient hematopoietic system are not comprehensively known and understood. Lethally irradiated mice can be rescued by adoptive transfer of very small numbers of lineage-depleted (Lin-), Sca1-positive cells, which reconstitute hematopoiesis. This competence of HSPCs to repopulate the hematopoietic system is often used as a measure of the functionality of HSPCs and to evaluate the contribution of individual proteins to this process.

In this concise report we demonstrate distinct and mostly antagonistic roles for the SWEF proteins in HSPC homeostasis and in reconstitution of the hematopoietic system.

## Materials and Methods

### Mice

Wild-type, *Swap-70*^*-/-*^ and *Def6*^*-/-*^ mice on the C57BL/6 background were previously described [7, 15]. Double knockout (DKO) *Swap-70*^*-/-*^*Def6*^*-/-*^ mice were generated from breeding of single *Swap-70*^*-/-*^ and *Def6*^*-/-*^. Animals were bred and maintained under pathogen-free conditions in the Animal Facility of the Medical Theoretical Center (MTZ) at the Medical Faculty of Technische Universität Dresden according to approved animal welfare guidelines and with approval of the State of Saxony (Az 24-9168-11-1/2011-13). The Institutional Animal Care and Use Committees (Tierschutzkommission der Technischen Universität Dresden und Tierschutzkommission des Landes Sachsen) specifically approved this study.

### FACS analysis and sorting

Staining of surface markers was performed according to standard procedures. Briefly, freshly isolated bone marrow (BM) cells were immunostained on ice in PBS, 2 mM EDTA in the presence of either mouse immunoglobulin G (IgG) whole molecule (1μg/ml, Jackson ImmunoResearch) or 2% v/v FCS to block non-specific binding through Fc receptors. Cells were incubated with different combinations of fluorescently labeled, purified or biotin-conjugated antibodies (Abs) for 25 min, followed by 20 min incubation with second-step reagents (secondary fluorescently labeled Abs or conjugated to streptavidin) when necessary. Antibodies were purchased from BD Bioscience (CD19 (clone 1D3) bio, CD11b (clone M1/70) bio, B220 (clone RA3-6B2) bio, c-kit (clone 2B8) PE-Cy7 and APC, Sca1 (clone D7) PE-Cy7 and FITC, eBioscience (Ter119 (clone Ter-119) bio, CD3e (clone 145-2C11) bio, CD16/32 (clone 93) PE-Cy7 and Pacific Blue, CD34 (clone RAM34) FITC, IL-7R〈 (clone A7R34) bio, Gr-1 (clone RB6-8C5) bio). Intracellular staining was carried out after staining of surface markers. Cells were fixed and permeabilized using a Cytofix/Cytoperm buffer (BD Bioscience). Then samples were incubated with primary and subsequently with secondary Abs at optimal concentrations that were experimentally standardized. For enrichment of lineage-negative cells BM was labeled with lineage Abs following labeling with magnetic beads (Miltenyi Biotec) and subjected to negative selection on MACS columns (Miltenyi Biotec). Flow cytometry analysis was carried out on a BD LSRII flow cytometer with FACSDiva software (BD Biosciences). Cells were sorted on FACSAria flow cytometer. Data were analyzed with FlowJo 6.1.1 software (Tristar).

For cell cycle analysis, cells were stained with the respective cell type-specific surface markers followed by fixation and permeabilization using Cytofix/Cytoperm solution (BD Bioscience) and incubation with Ki67 (clone SolA15) PE-Cy7 (eBioscience) and DAPI (Sigma Aldrich) for 30 min.

Analysis of apoptosis was performed using an Annexin V Apoptosis Detection Set (eBioscience) and DAPI according to the manufacturer`s recommendations. In brief, following cell surface staining, cells were washed with binding buffer and stained with Annexin V PE-Cy7 and DAPI for 15 min at room temperature.

### PCR

Total RNA was isolated from 2.5x10^4^-5x10^5^ sorted lineage-negative, kit-positive, Sca-positive (LSK) cells using a RNeasy Micro kit (Qiagen). First-strand cDNA was synthesized from 70 ng of RNA using SuperScript II Reverse Transcriptase (Invitrogen). DEF6 PCR primers were: 5´ CAACGTGAAACACTGGAATGTC; 5´ CTGAGAGGGTTCTGTTACCTTG; product size– 176 bp. GAPDH primers were: 5´ GAGAAACCTGCCAAGTATGAC; 5’ GGAGTTGCTATGAAGTTGC.

### Adoptive cell transfer

Wild-type recipient mice were lethally irradiated (9 Gy) and were given an intravenous injection of 1:1 mixture (5x10^3^ in total) of FACS sorted LSK from 2 donors in different combinations (wt and *Swap-70*^*-/-*^, wt and *Def6*^*-/-*^, wt and *SWAP-70*^*-/-*^*Def6*^*-/-*^). Together with LSK mixture recipients received 2x10^5^ of total wt BM cells. Donor and recipient mice differed in congenic markers (CD45.1, CD45.2 and CD45.1.2) for further discrimination of progeny by FACS analysis. Animals were kept in pathogen-free conditions during the period of recovery and received neomycin water for 14 days. Blood was analyzed at desired time points.

### Statistic analysis

Data were analyzed using unpaired two-tailed *t*-test. *P<0.05, **P<0.01, ***P<0.005.

## Results and Discussion

### SWAP-70 and DEF6 expression in hematopoietic stem and precursor cells

**Fig 1:**
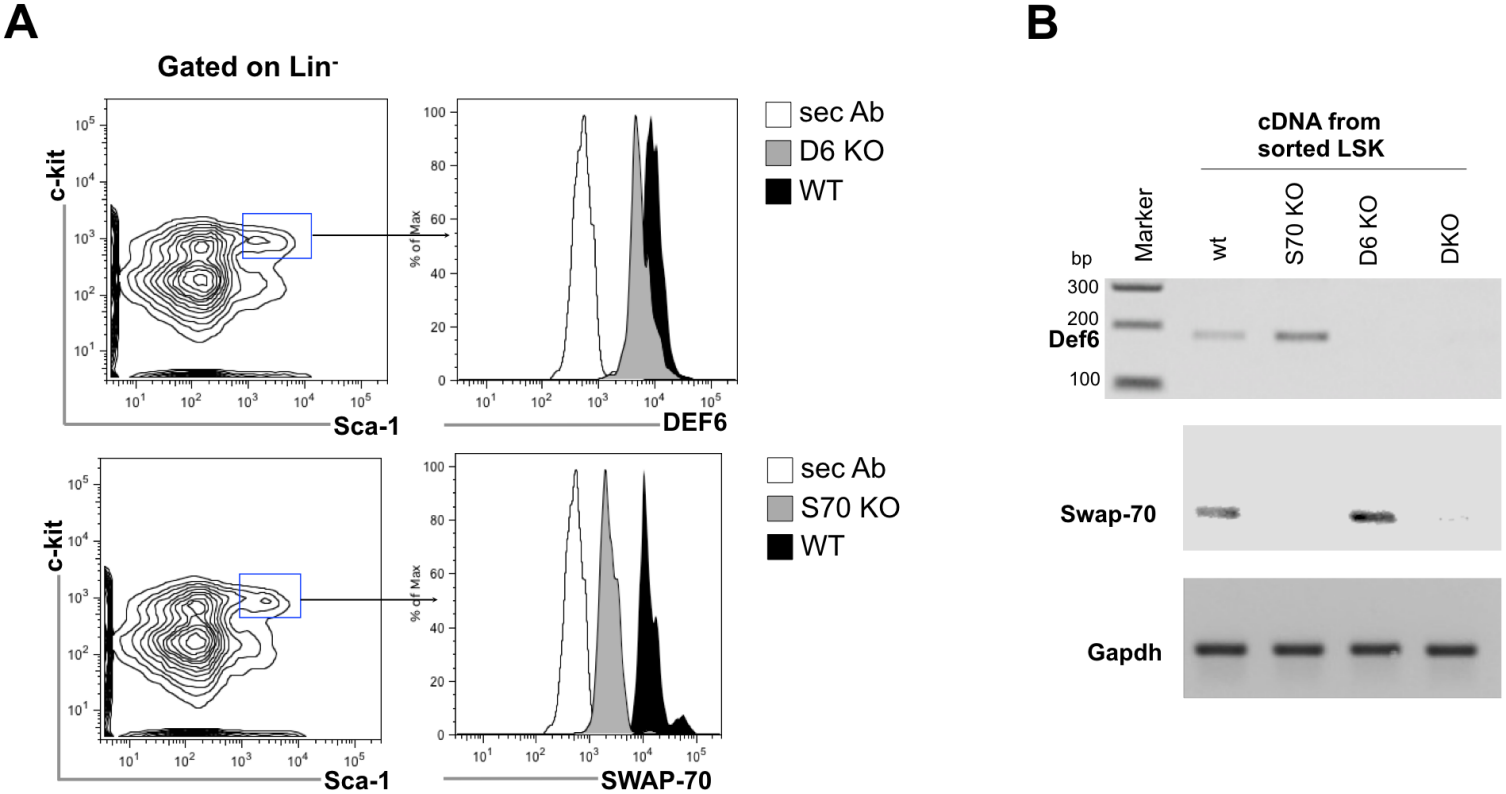
SWAP-70 and DEF6 expression analysis in hematopoietic stem/progenitor cells. **(A)** Intracellular FACS staining for DEF6 (upper dot plots) and SWAP-70 (lower dot plots) in BM cells. Left: lineage-negative cells, right: HSCs and early progenitors, subgated Lin^-^c-kit^+^Sca-1^+^. Data shown are representative of two independent experiments. **(B)** mRNA expression analysis of *Def6* (product: 176 bp) in sorted LSK cells from wild type (WT), *SWAP-70*^*-/-*^(S70 KO, *Def6*^*-/-*^(D6 KO) and *SWAP-70*^*-/-*^*Def6*^*-/-*^ (DKO) mice. *Gapdh* (product: 100 bp) and an unspecific PCR product (380 bp) served as controls.

To assess the putative role of SWAP-70 and DEF6 in mouse HSPC biology and hematopoiesis we first determined the expression of the two SWEF proteins in HSPCs. Intracellular FACS staining of total bone marrow (BM) cells was performed using previously described, affinity-purified rabbit polyclonal anti-SWAP-70 antibodies [12] and anti-DEF6 rabbit polyclonal antibodies [15]. The presence of SWAP-70 protein in HSC as determined by intracellular FACS staining was earlier shown by us [16]. Both proteins were detected in the LSK (Lineage-negative, Sca1-positive, c-Kit-positive) fraction of wild-type (wt) mice but not of mice deficient in either of these proteins (Fig 1A).

We also detected DEF6 mRNA expression by RT-PCR in FACS-sorted LSK cells from lineage-depleted (Lin^-^) BM from wt and *Swap-70*^*-/-*^ mice but not from *Def6*^*-/-*^, and *Swap-70*^*-/-*^*Def6*^*-/-*^ (DKO) mice (Fig 1B). Similarly, Swap-70 mRNA was detected in LSK cells from wt and *Def6*^*-/-*^ mice. Thus, both SWEF proteins are expressed in HSPCs.

### SWAP-70 and DEF6 deficiencies perturb HSPC numbers

**Fig 2:**
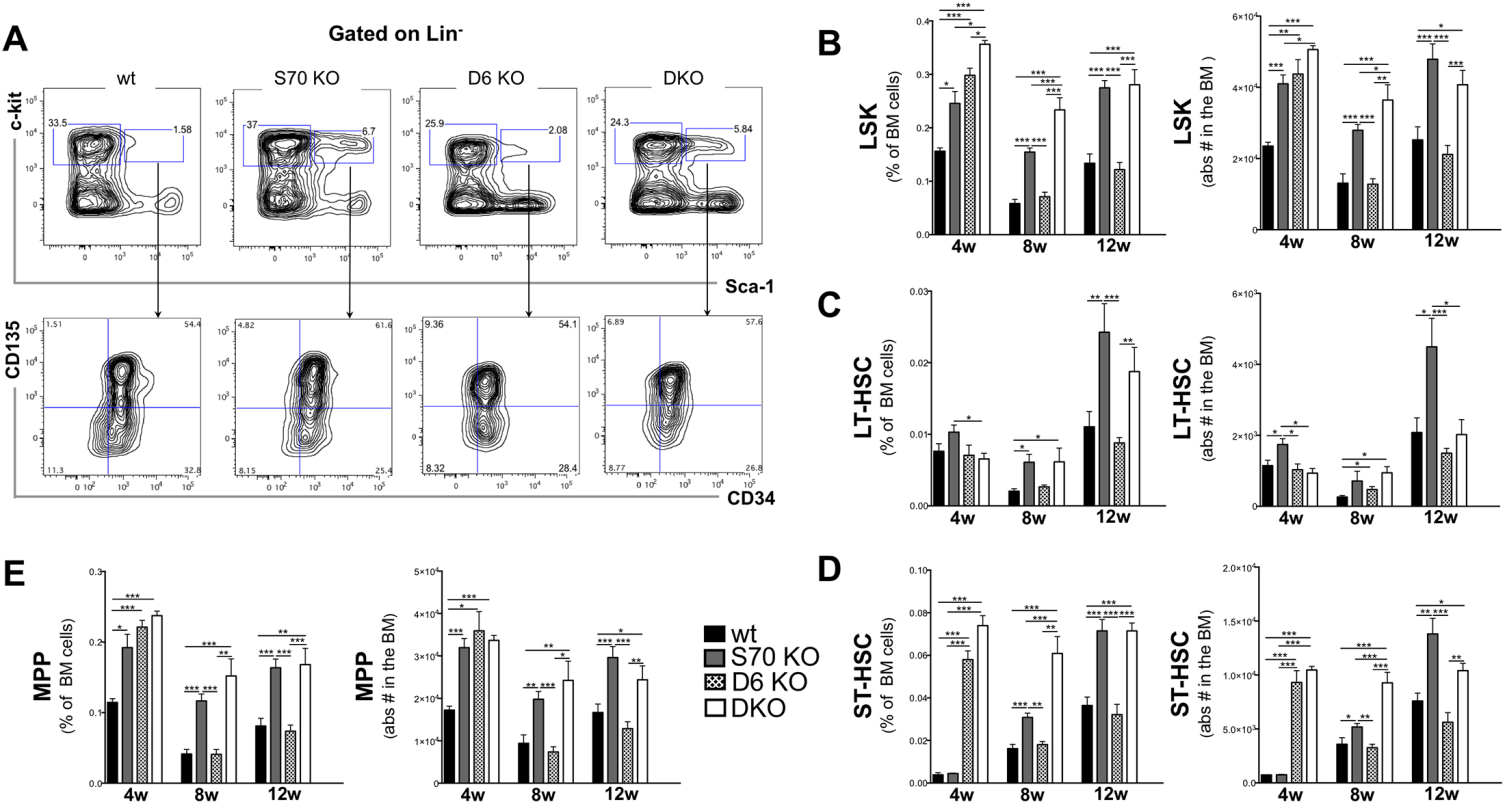
Hematopoietic stem cell and progenitor analysis in the bone marrow of *Swap-70*^*-/-*^, *Def6*^*-/-*^ and double knock-out mice. (A) Representative FACS plots of freshly isolated BM from wt, *Swap-70*^*-/-*^ (S70 KO), *DEF6*^*-/-*^ (D6 KO) and *Swap-70*^*-/-*^*DEF6*^*-/-*^ (DKO) 8-week old mice. Upper plots: subgated DAPI^-^Lin^-^ BM cells. Percentages shown are of parental gate. Lower plots: subgated DAPI^-^Lin^-^kit^+^Sca^+^ (LSK) to discriminate long-term HSC (LT-HSC, CD34^-^CD135^-^), short-term HSC (ST-HSC, CD34^+^CD135^-^) and multiple progenitors (MPP, CD34^+^CD135^+^). Percentages shown are of parent LSK populations. **(B)** Relative (on the left) and absolute (on the right) LSK cell numbers in the BM of wt (black bars), SWAP-70 (grey bars) and DEF6 (dotted bars) single and DKO (white bars) of different ages (4, 8 and 12 weeks old) quantified by FACS. **(C)** Relative (on the left) and absolute (on the right) LT-HSC cell numbers in the BM of wt, SWAP-70 and DEF6 single and DKO of different ages quantified by FACS. **(D)** Relative (on the left) and absolute (on the right) ST-HSC cell numbers in the BM of wt (black bars), SWAP-70 (grey bars) and DEF6 (dotted bars) single and DKO (white bars) of different ages (4, 8 and 12 weeks old) quantified by FACS. **(E)** Relative (on the left) and absolute (on the right) MPP cell numbers in the BM of wt (black bars), SWAP-70 (grey bars) and DEF6 (dotted bars) single and DKO (white bars) of different ages (4, 8 and 12 weeks old) quantified by FACS. At least 5 mice of each genotype per group were analyzed, >8 mice 12 weeks of age were analyzed. Mean ± SD. *P<0.05, **P<0.01, ***P<0.005.

To investigate the potential role of both proteins in HSPCs, the frequencies and numbers of HSPCs, committed progenitors, and mature hematopoietic cell types were determined. We analyzed BM from wt, *Swap-70*^*-/-*^, *Def6*^*-/-*^, and *Swap-70*^*-/-*^*Def6*^*-/-*^ mice of different ages (4 to 12 weeks after birth) and observed an increase in the relative as well as absolute number of LSK cells in the absence of SWAP-70 and of both proteins (Fig 2A and 2B). This increase was more pronounced in 8-week-old (~2.5-fold) and in 12-week-old (~2.0-fold) mice than in 4-week-old (~1.6-fold) mice, but was statistically significant at every time point. Thus, without SWAP-70 HSPCs accumulate in the BM suggesting a failure to further develop and/or to migrate between the respective niches. DEF-6 deficiency caused an ~2-fold increase in LSK numbers at 4 weeks of age, after which this increase was not observed anymore (8 weeks), but the LSK number rather slightly decreased (12 weeks) (Fig 2A and 2B). Thus, while in absence of SWAP-70 the LSK population accumulates at all time points, a similar increase upon DEF6 deficiency is not maintained with increasing age. The DKO LSK numbers reveal a dominant SWAP-70-dependent phenotype.

Next we sub-gated the LSK fraction into long-term (LT-HSC), short-term hematopoietic stem cells (ST-HSC), and multiple progenitor cells (MPP) according to surface expression of CD34 and flt3 (CD135) [17]. In 4-week old mice the number of LT-HSCs was not significantly higher when SWAP-70 was absent (Fig 2A and 2C). A significant increase in LT-HSCs from *Swap-70*^*-/-*^ mice was observed at 8 weeks (~3-fold) and 12 weeks (~2.5-fold) of age. This pattern was largely reproduced in DKO animals. *Def6*^*-/-*^ mice showed wt-like LT-HSC numbers at all age groups analyzed, and a slight decrease at 12 weeks of age. Similarly, the ST-HSC compartment of *Swap-70*^*-/-*^ mice showed significantly higher cell numbers, up to 2-fold, in mice of 8 and 12 weeks compared to wt (Fig 2D). In contrast, the absence of DEF6 did only affect ST-HSC numbers at the age of 4 weeks showing an increase, which was not maintained. This may reflect the early increase of LSKs at 4 weeks in this mutant. However, the DKO showed increased ST-HSC numbers in at all age groups analyzed. The effect of SWAP-70 or DEF6 deficiency on the numbers of MPP was very similar to their effect on LSK cell numbers (Fig 2E).

Generally, the numbers of LSK, LT-HSC and ST-HSC decreased from 4 weeks to 8 weeks of age in wt, *Swap-70*^*-/-*^ and *Def6*^*-/-*^ mice. The most prominent effect of the mutants was the accumulation of LSK cells, LT-HSCs, ST-HSCs and MPPs in the *Swap-70*^*-/-*^ mice at 12 weeks of age compared to wt animals. This suggests that SWAP-70 and to a lesser extent DEF6 control HSPC balance.

**Fig 3:**
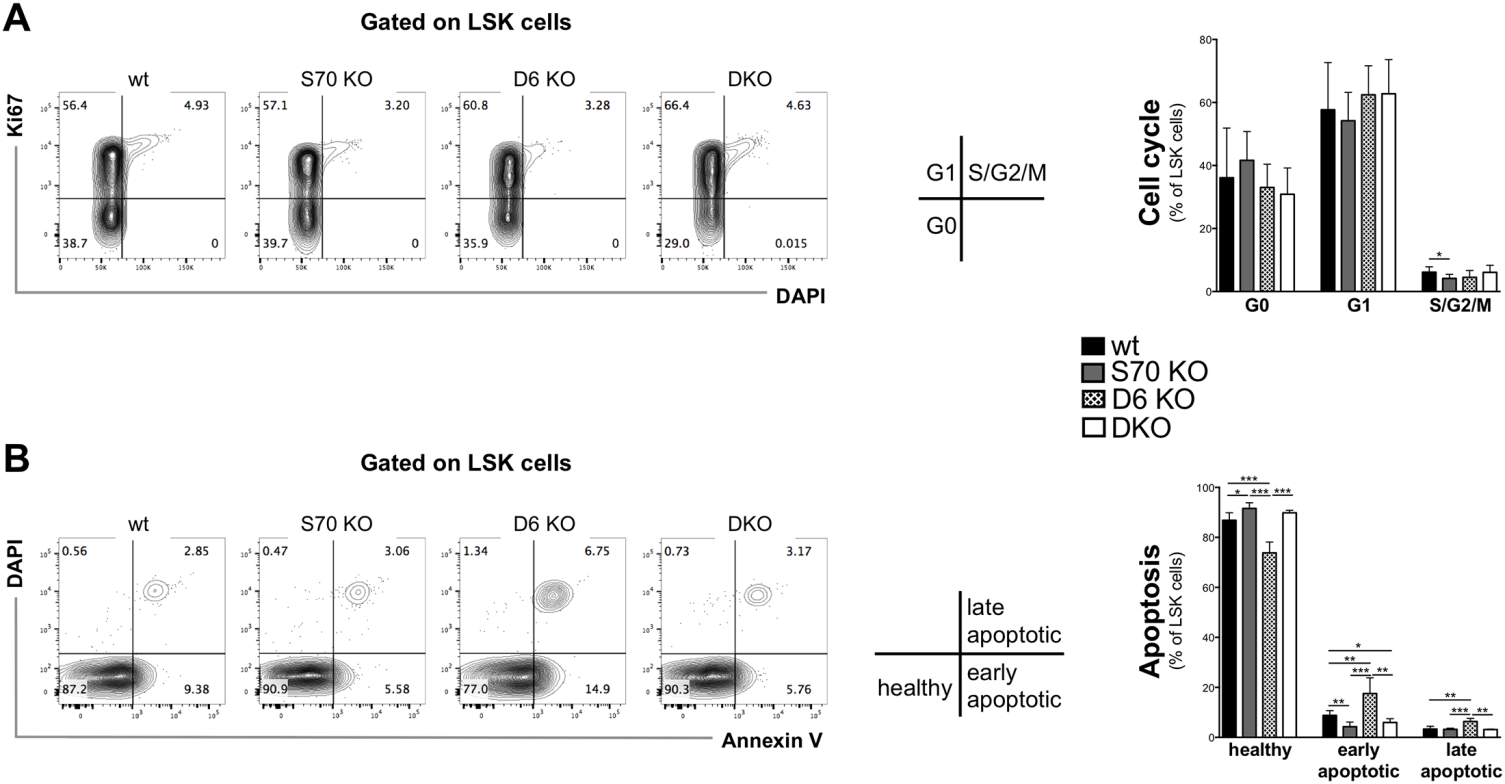
Effects of SWAP-70- and DEF-6-deletion on cycling and apoptosis of LSK cells. **(A)** Representative flow cytometric analysis of DNA content (DAPI) and intracellular Ki-67 expression on gated Lin^-^kit^+^Sca^+^ LSK cells derived from 12-week-old wt, *Swap-70*^*-/-*^ (S70 KO), *Def6*^*-/-*^ (D6 KO) and *Swap-70*^*-/-*^*Def6*^*-/-*^ (DKO) mice. The percentage of cells in the respective cell cycle stage is indicated next to the corresponding gate. Graph depicts mean percentage of LSK cells that are in G0, G1 or S/G2/M phase of the cell cycle. (**B**) Representative flow cytometric analysis of apoptosis in LSK cells detected with Annexin V and DAPI. Graph displays mean percentage of Annexin V+ DAPI- early apoptotic and Annexin V+ DAPI+ late apoptotic LSK cells (n = 4–6 mice per genotype).

To address the effect of the SWEF proteins on stem cell proliferation, we used Ki67 and DAPI labeling to determine the cell cycle status of LSK cells in 12-week-old wt, *Swap-70*^*-/-*^, *Def6*^*-/-*^, and *Swap-70*^*-/-*^*Def6*^*-/-*^ mice. These analyses revealed no major differences in the cell cycle phase distribution between LSK cells of the four genotypes examined (Fig 3A). However, analysis of apoptosis revealed that a significantly lower number of SWAP-70-deficient LSK cells were undergoing apoptosis. In contrast, the absence of DEF6 led to significantly higher numbers of apoptotic cells (Fig 3B), suggesting that SWAP-70-deficiency suppresses apoptosis in HSC, causing an accumulation of these cells in the bone marrow as described above, while DEF6-deficiency promotes apoptosis in HSC, leading to the slight decrease of HSC.

### Development of committed progenitors is compromised in absence of SWAP-70 and DEF6

**Fig 4:**
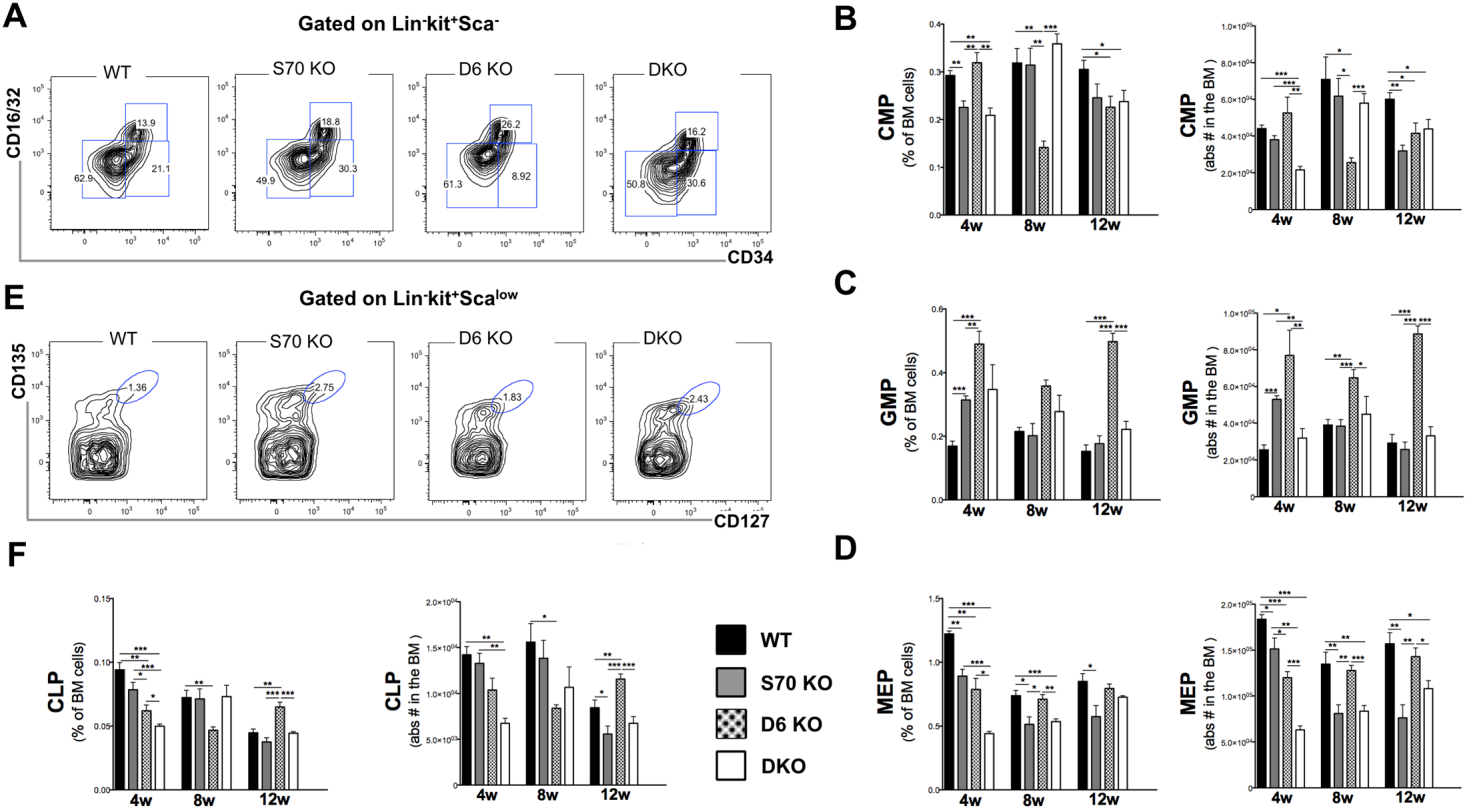
Lymphoid and myeloid progenitor analysis in the bone marrow of *Swap-70*^*-/-*^, *DEF6*^*-/-*^ and double knock-out mice. **(A)** and **(E)** Representative FACS plots of freshly isolated BM from wt, *Swap-70*^*-/-*^ (S70 KO), *Def6*^*-/-*^ (D6 KO) and *Swap-70*^*-/-*^*Def6*^*-/-*^ (DKO) 8-week old mice. Upper plots (A) subgated DAPI^-^Lin^-^kit^+^Sca^-^ BM cells to discriminate common myeloid progenitors (CMP, CD34^+^CD16/32^-^), granulocyte-monocyte progenitors (GMP, CD34^+^CD16/32^+^) and megakaryocyte-erythroid progenitors (MEP, CD34^-^CD16/32^-^). Lower plots (E): subgated DAPI^-^Lin^-^kit^+^Sca^low^ to discriminate common lymphoid progenitors (CLP, CD135^+^CD135^+?^). Percentages shown are of parental gate. **(B)** Relative (on the left) and absolute (on the right) CMP cell numbers in the BM of wt (black bars), SWAP-70 (grey bars) and DEF6 (dotted bars) single and DKO (white bars) of different ages (4, 8 and 12 weeks old) quantified by FACS. **(C)** Relative (on the left) and absolute (on the right) GMP cell numbers in the BM of wt, SWAP-70 and DEF6 single and DKO of different ages quantified by FACS. **(D)** Relative (on the left) and absolute (on the right) MEP cell numbers in the BM of wt (black bars), SWAP-70 (grey bars) and DEF6 (dotted bars) single and DKO (white bars) of different ages (4, 8 and 12 weeks old) quantified by FACS. **(E)** Relative (on the left) and absolute (on the right) CLP cell numbers in the BM of wt (black bars), SWAP-70 (grey bars) and DEF6 (dotted bars) single and DKO (white bars) of different ages (4, 8 and 12 weeks old) quantified by FACS. At least 4 mice of each genotype per group were analyzed, in many instances >10 mice were analyzed. Mean ± SD. *P<0.05, **P<0.01, ***P<0.005.

Analyses of progenitor cells committed to the myeloid or lymphoid lineages (Fig 4A and 4E) showed that the numbers of common myeloid progenitors (CMP) are differently affected by the absence of SWAP-70 or DEF6 or both (Fig 4B). SWAP-70 deficiency caused a decrease of CMPs at 4 weeks and 12 weeks of age. The difference at 8 weeks was not statistically significant. Absence of DEF6 led to a decrease at 8 and 12 weeks of age. In the DKO, a decrease of about 2-fold (absolute numbers) was seen but not maintained at 8 and 12 weeks of age, where the decrease was more moderate at about 30%.

The frequency and the number of granulocyte-monocyte precursors (GMPs) was strongly affected by the lack of DEF6. *Def6*^*-/-*^ mice had up to 3-fold more GMPs in all age groups. *Swap-70*^*-/-*^ mice showed twice as many GMPs as the wt only at 4 weeks of age. Wt and DKO mice did not significantly differ in GMP numbers at all three time points, indicating a dominant effect of the SWAP-70 deficiency (Fig 4C). Thus, in absence of DEF6, SWAP-70 de-regulates GMP numbers, and this effect is alleviated by removal of SWAP-70.

Megakaryocyte-erythrocyte progenitors (MEPs) were only slightly reduced by the lack of SWAP-70 or DEF6 in 4 weeks old mice. The simultaneous elimination of both proteins resulted in more than 2-fold decrease in MEP numbers indicating synergistic activity of the SWEF proteins at this time point. At the ages of 8 and 12 weeks relative as well as absolute MEP numbers mainly depended on SWAP-70, whose absence either alone or together with DEF6 caused reduced numbers. *Def6*^*-/-*^ mice showed wt-like numbers at 8 and 12 weeks of age (Fig 4D).

Similarly to MEPs, the numbers of common lymphoid progenitor (CLP) cells were reduced by ~2-fold in 4 weeks-old DKO mice. SWAP-70 deficiency alone did not affect the CLP numbers in mice of the three age groups. In contrast, *Def6*^*-/-*^ mice showed the lowest CLP numbers of all genotypes at 4 and 8 weeks, a trend that was reversed at 12 weeks. Such reversals of initial phenotypes in young mice may be the result of compensatory processes within the ageing mutant animals (Fig 4F).

Together, these data show that DEF6 and SWAP-70 play antagonistic roles in GMP development and moderately agonistic roles in MEP differentiation. Only DEF6 deficiency caused a decrease in CLP.

### Development of B lymphocytes and myeloid cells is not affected by SWEF deficiency, but T lymphocytes are reduced

**Fig 5:**
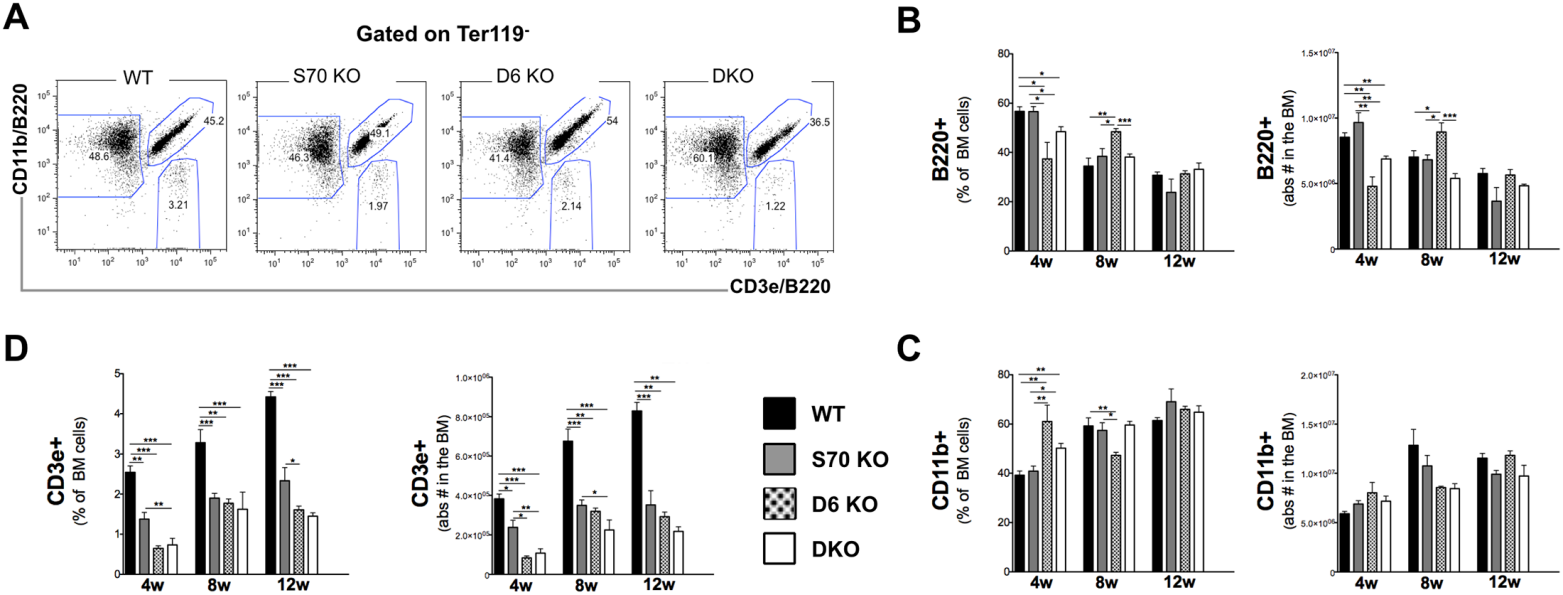
B-lymphocyte (B220^+^), T-lymphocyte (CD3e^+^) and myeloid (CD11b^+^) cell analysis in the bone marrow of *Swap-70*^*-/-*^, *DEF6*^*-/-*^ and DKO mice. **(A)** Representative FACS plots of freshly isolated BM from wt, *Swap-70*^*-/-*^ (S70 KO), *DEF6*^*-/-*^ (D6 KO) and *Swap-70*^*-/-*^*DEF6*^*-/-*^ (DKO) 8-week old mice. Total BM was subgated to discriminate B-lymphocytes (B220^+^), T-lymphocytes (CD3e^+^) and myeloid (CD11b^+^) cells. **(B)** Relative (on the left) and absolute (on the right) B220^+^ cell numbers in the BM of wt (black bars), SWAP-70 (grey bars) and DEF6 (dotted bars) single and DKO mice (white bars) of different ages (4, 8 and 12 weeks old) quantified by FACS. **(C)** Relative (on the left) and absolute (on the right) CD11b^+^ cell numbers in the BM of wt, SWAP-70 and DEF6 single and double knock-out mice of different ages quantified by FACS. **(D)** Relative (on the left) and absolute (on the right) CD3e^+^ cell numbers in the BM of wt (black bars), SWAP-70 (grey bars) and DEF6 (dotted bars) single and DKO (white bars) of different ages (4, 8 and 12 weeks old) quantified by FACS. At least 4 mice of each genotype per group were analyzed, in many instances >10 mice were analyzed. Mean ± SD. *P<0.05, **P<0.01, ***P<0.005.

In the mature cell compartment of the bone marrow the numbers of cells positive for the B lineage marker B220 were slightly decreased in *Def6*^*-/-*^ and DKO mice of 4 weeks of age as compared to wt and *Swap-70*^*-/-*^ mice, but there were small increases or no differences at 8 and 12 weeks of age, respectively. *Swap-70*^*-/-*^ and DKO mice showed no differences to wt (Fig 5A and 5B). Thus, the aberrations in numbers of HSPCs and of more committed precursors observed in bone marrow of the mutant mice did not cause significant shifts in numbers of B lymphocytes, probably because the niches were filled over time. This is independent of the deficiencies that individual peripheral B cell populations show in their further maturation and/or in response to challenges as reported previously [7, 18, 19]. The absolute numbers of CD11b+ myeloid cells were not significantly affected by either single or double deficiency (Fig 5A and 5C).

In contrast, the numbers of CD3e-positive T cells in the BM were strongly affected by either single deficiency and in the DKO. The BM of *Swap-70*^*-/-*^, *Def6*^*-/-*^ and DKO mice showed about half of wt cell numbers in both, relative and absolute terms, and in mice of all ages analyzed. The most drastic decrease was observed in *Def6*^*-/-*^ and DKO mice at 4 weeks of age, where the CD3e+ numbers were reduced 4-fold. While in wt the CD3e+ cell numbers increased with age, there was almost no increase in the three mutant strains (Fig 5A and 5D).

Bone marrow can function as a major reservoir for memory T cells. After their generation in secondary lymphoid organs, memory CD3+ T cells preferentially locate to the bone marrow for homeostatic maintenance, where they reside in specified niches for long periods of time [20, 21]. The prominent reduction of the CD3+ T cell population within the bone marrow of the mutant mice could be a result of intrinsic T cell defects. It is less likely to be an effect of the altered bone marrow environment, which is osteopetrotic only in Swap-70^-/-^ mice [22]. In contrast to SWAP-70, DEF6 is expressed in thymocytes and peripheral T cells and has been shown to be an important regulator of T cell development and homeostasis [4, 5, 23]. Furthermore, a role of DEF6 in memory T cell expansion has been proposed [24], but an impact of DEF6 on memory T cell maintenance in the bone marrow has not yet been described. It needs to be determined in more detailed studies, which factors contribute to the observed decrease of CD3+ T cells in the bone marrow of *Swap-70*^*-/-*^, *Def6*^*-/-*^ and DKO mice and whether this is caused by defective recruitment or maintenance of these putative memory T cells.

### The function of SWAP-70 and DEF6 is intrinsic to HSC/HSPCs

**Fig 6:**
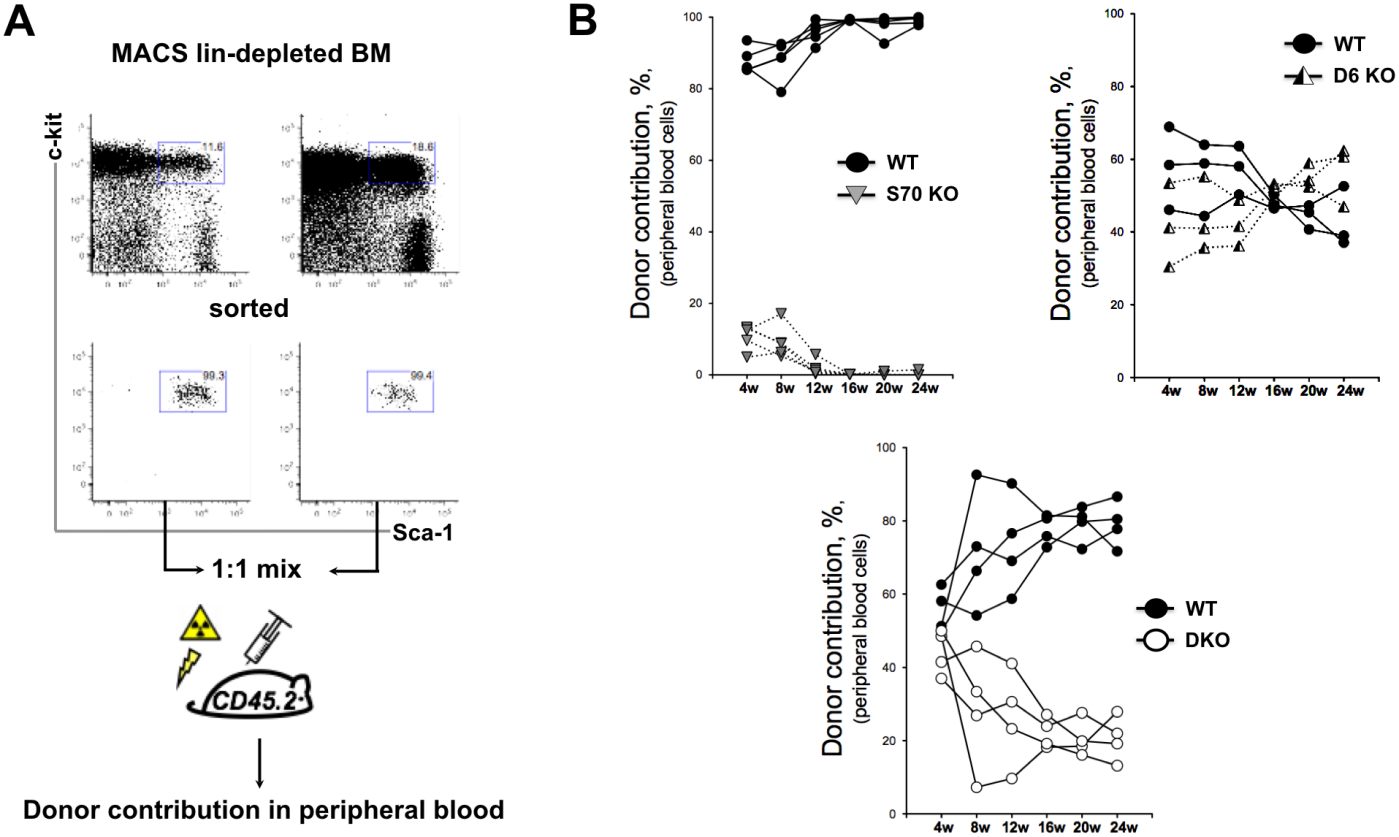
Analysis of donor contribution in *de novo* generated hematopoietic cells upon competitive LSK cell transfer into lethally irradiated wt recipients. (A) Experimental set-up of competitive LSK transfer. Freshly isolated BM cells from donor mice carrying congenic CD45.2 and CD45.1.2 markers were lineage-depleted by MACS and subsequently FACS sorted to get the pure LSK cells. Lethally irradiated recipients received 1:1 mixture of donor competitor cells. Peripheral blood reconstitution was monitored by FACS every 4 weeks after transplantation. (B) Kinetics of donor contribution into peripheral blood cell generation upon competitive transfer of wt and *Swap-70*^*-/-*^ LSK on the left, wt and *DEF6*^*-/-*^ LSK on the right, and wt and *Swap-70*^*-/-*^*DEF6*^*-/-*^ LSK on the bottom.

Since both proteins are expressed in a variety of hematopoietic cell types, and since *Swap-70*^*-/-*^ but not *Def6*^*-/-*^ mice develop mild osteopetrosis [22], some of the variations and age-dependent compensatory mechanisms described above may have been generated through interactions of the HSPCs with the mutant bone and marrow environment. Therefore we asked whether the role of SWAP-70 and DEF6 in HSC maintenance and differentiation is intrinsic to these cells. We transplanted sorted HSC/HSP cells from *Swap-70*^*-/-*^, *Def6*^*-/-*^ or DKO into lethally irradiated wt recipients. The sorted LSK cells were competitively transferred in a ratio of wt: mutant of 1:1. The use of the congenic markers CD45.1 and CD45.2 allowed distinguishing, wt and mutant donor and recipient cell populations (Fig 6A and 6B).

Donor contributions to the generation of peripheral blood cells were monitored for 24 weeks. We observed a very strong requirement for SWAP-70 in reconstitution of the hematopoietic system. The *Swap-70*^*-/-*^ LSK cells were not able to compete with wt LSK cells. *Swap-70*^*-/-*^ progeny was hardly detectable in the recipient blood at any time point after 12 weeks up to 24 weeks after transfer (and in an extended experiment up to 39 weeks post transfer; data not shown). The *Swap-70*^*-/-*^ cells that were initially detectable although only at 10% of wt levels, disappeared at 12 weeks after transfer.

In contrast, transferred *Def6*^*-/-*^ LSK cells efficiently contributed to the *de novo* blood cell generation and after 20 weeks the recipient mice showed an even slightly higher proportion of cells generated from DEF6-deficient HSCs. Four weeks post transplantation the recipients transplanted with wt and DKO HSC/HPCs showed an equal contribution of the donors to the peripheral blood cells. However throughout the following weeks the capacity of DKO HSC/HPCs to compete against wt HSC/HPCs decreased, and about 75% of blood cells were generated by wt donor cells. Despite this low reconstitution efficiency, the DKO HSC/HPCs generated substantially more peripheral blood cells than the *Swap-70*^*-/-*^ donor cells.

Together, we revealed distinct and largely antagonistic roles of the two SWEF proteins in HSPC biology. In one instance only we observed synergistic effect of the two deficiencies, i.e. in the decreases in MEPs in young mice. DEF6 deficiency alone had a prominent effect in only one case, the numbers of CLPs at 4 and 8 weeks of age. Otherwise, the phenotypes observed upon SWAP-70 deficiency are in most of the cases dominant: HSPCs accumulate in *Swap-70*^*-/-*^ and DKO mice, but not in *Def6*^*-/-*^ animals; the increase in GMPs upon DEF deficiency was abolished by additional SWAP-70 deficiency; the failure to rescue hematopoiesis upon HSPC transfer was seen in *Swap-70*^*-/-*^ and DKO mice, but not in *Def6*^*-/-*^ animals. Here, the antagonism of the two proteins became apparent, since DEF6 deficiency rescued some of the reconstitution competence of SWAP-70 deficient HSPCs. Thus, in absence of SWAP-70, DEF6 contributes to the complete failure of HSPCs to reconstitute the hematopoietic system. This also suggests that SWAP-70’s activity itself is required only for about 75% of HSPC competence. It will be important in the future to determine the opposing pathways, with which the two SWEF proteins control and balance HSPC biology.

## References

[1] Borggrefe T, Wabl M, Akhmedov AT, Jessberger R. A B-cell-specific DNA recombination complex. J Biol Chem. 1998;273(27):17025–35. Epub 1998/06/27. . 9642267 10.1074/jbc.273.27.17025

[2] Hotfilder M, Baxendale S, Cross MA, Sablitzky F. Def-2, -3, -6 and -8, novel mouse genes differentially expressed in the haemopoietic system. Br J Haematol. 1999;106(2):335–44. . 10460589 10.1046/j.1365-2141.1999.01551.x

[3] Shinohara M, Terada Y, Iwamatsu A, Shinohara A, Mochizuki N, Higuchi M, et al SWAP-70 is a guanine-nucleotide-exchange factor that mediates signalling of membrane ruffling. Nature. 2002;416(6882):759–63. Epub 2002/04/19. 10.1038/416759a 416759a [pii]. . 11961559

[4] Gupta S, Lee A, Hu C, Fanzo J, Goldberg I, Cattoretti G, et al Molecular cloning of IBP, a SWAP-70 homologous GEF, which is highly expressed in the immune system. Hum Immunol. 2003;64(4):389–401. . 12651066 10.1016/s0198-8859(03)00024-7

[5] Tanaka Y, Bi K, Kitamura R, Hong S, Altman Y, Matsumoto A, et al SWAP-70-like adapter of T cells, an adapter protein that regulates early TCR-initiated signaling in Th2 lineage cells. Immunity. 2003;18(3):403–14. . 12648457 10.1016/s1074-7613(03)00054-2

[6] Ihara S, Oka T, Fukui Y. Direct binding of SWAP-70 to non-muscle actin is required for membrane ruffling. J Cell Sci. 2006;119(Pt 3):500–7. Epub 2006/01/19. jcs.02767 [pii] 10.1242/jcs.02767 . 16418221

[7] Pearce G, Angeli V, Randolph GJ, Junt T, von Andrian U, Schnittler HJ, et al Signaling protein SWAP-70 is required for efficient B cell homing to lymphoid organs. Nat Immunol. 2006;7(8):827–34. Epub 2006/07/18. ni1365 [pii] 10.1038/ni1365 . 16845395

[8] Wakamatsu I, Ihara S, Fukui Y. Mutational analysis on the function of the SWAP-70 PH domain. Molecular and cellular biochemistry. 2006;293(1–2):137–45. . 16786189 10.1007/s11010-006-9236-1

[9] Ocana-Morgner C, Wahren C, Jessberger R. SWAP-70 regulates RhoA/RhoB-dependent MHCII surface localization in dendritic cells. Blood. 2009;113(7):1474–82. Epub 2008/09/20. blood-2008-04-152587 [pii] 10.1182/blood-2008-04-152587 . 18802007

[10] Pearce G, Audzevich T, Jessberger R. SYK regulates B-cell migration by phosphorylation of the F-actin interacting protein SWAP-70. Blood. 2011;117(5):1574–84. Epub 2010/12/03. blood-2010-07-295659 [pii] 10.1182/blood-2010-07-295659 . 21123826

[11] Biswas PS, Gupta S, Stirzaker RA, Kumar V, Jessberger R, Lu TT, et al Dual regulation of IRF4 function in T and B cells is required for the coordination of T-B cell interactions and the prevention of autoimmunity. J Exp Med. 2012;209(3):581–96. Epub 2012/03/01. jem.20111195 [pii] 10.1084/jem.20111195 22370718 PMC3302237

[12] Chacon-Martinez CA, Kiessling N, Winterhoff M, Faix J, Muller-Reichert T, Jessberger R. The switch-associated protein 70 (SWAP-70) bundles actin filaments and contributes to the regulation of F-actin dynamics. J Biol Chem. 2013;288(40):28687–703. Epub 2013/08/08. M113.461277 [pii] 10.1074/jbc.M113.461277 23921380 PMC3789966

[13] Becker AJ, Mc CE, Till JE. Cytological demonstration of the clonal nature of spleen colonies derived from transplanted mouse marrow cells. Nature. 1963;197:452–4. Epub 1963/02/02. . 13970094 10.1038/197452a0

[14] Spangrude GJ, Heimfeld S, Weissman IL. Purification and characterization of mouse hematopoietic stem cells. Science. 1988;241(4861):58–62. Epub 1988/07/01. . 2898810 10.1126/science.2898810

[15] Chen Q, Gupta S, Pernis AB. Regulation of TLR4-mediated signaling by IBP/Def6, a novel activator of Rho GTPases. J Leukoc Biol. 2009;85(3):539–43. Epub 2008/12/17. jlb.0308219 [pii] 10.1189/jlb.0308219 19074553 PMC2653944

[16] Ripich T, Jessberger R. SWAP-70 regulates erythropoiesis by controlling {alpha}4 integrin. Haematologica. 2011 Epub 2011/09/02. haematol.2011.050468 [pii] 10.3324/haematol.2011.050468 . 21880631 PMC3232255

[17] Yang L, Bryder D, Adolfsson J, Nygren J, Mansson R, Sigvardsson M, et al Identification of Lin(-)Sca1(+)kit(+)CD34(+)Flt3- short-term hematopoietic stem cells capable of rapidly reconstituting and rescuing myeloablated transplant recipients. Blood. 2005;105(7):2717–23. Epub 2004/12/02. 2004-06-2159 [pii] 10.1182/blood-2004-06-2159 . 15572596

[18] Chopin M, Quemeneur L, Ripich T, Jessberger R. SWAP-70 controls formation of the splenic marginal zone through regulating T1B-cell differentiation. Eur J Immunol. 2010 Epub 2010/11/13. 10.1002/eji.201040556 21108474

[19] Quemeneur L, Angeli V, Chopin M, Jessberger R. SWAP-70 deficiency causes high-affinity plasma cell generation despite impaired germinal center formation. Blood. 2008;111(5):2714–24. Epub 2007/12/21. blood-2007-07-102822 [pii] 10.1182/blood-2007-07-102822 . 18094331 PMC2254552

[20] Tokoyoda K, Zehentmeier S, Hegazy AN, Albrecht I, Grun JR, Lohning M, et al Professional memory CD4+ T lymphocytes preferentially reside and rest in the bone marrow. Immunity. 2009;30(5):721–30. Epub 2009/05/12. S1074-7613(09)00187-3 [pii] 10.1016/j.immuni.2009.03.015 . 19427242

[21] Mazo IB, Honczarenko M, Leung H, Cavanagh LL, Bonasio R, Weninger W, et al Bone marrow is a major reservoir and site of recruitment for central memory CD8+ T cells. Immunity. 2005;22(2):259–70. Epub 2005/02/23. S1074-7613(05)00034-8 [pii] 10.1016/j.immuni.2005.01.008 . 15723813

[22] Garbe AI, Roscher A, Schuler C, Lutter AH, Glosmann M, Bernhardt R, et al Regulation of bone mass and osteoclast function depend on the F-actin modulator SWAP-70. J Bone Miner Res. 2012 Epub 2012/06/01. 10.1002/jbmr.1670 . 22648978

[23] Becart S, Charvet C, Canonigo Balancio AJ, De Trez C, Tanaka Y, Duan W, et al SLAT regulates Th1 and Th2 inflammatory responses by controlling Ca2+/NFAT signaling. J Clin Invest. 2007;117(8):2164–75. Epub 2007/07/28. 10.1172/JCI31640 17657315 PMC1924495

[24] Feau S, Schoenberger SP, Altman A, Becart S. SLAT regulates CD8+ T cell clonal expansion in a Cdc42- and NFAT1-dependent manner. J Immunol. 2013;190(1):174–83. Epub 2012/12/01. jimmunol.1201685 [pii] 10.4049/jimmunol.1201685 23197258 PMC3529792

